# Expression of MK-1 and RegⅣ and its clinicopathological significances in the benign and malignant lesions of gallbladder

**DOI:** 10.1186/1746-1596-6-100

**Published:** 2011-10-21

**Authors:** Leping Yang, Sigen Lan, Jieqiong Liu, Zhulin Yang

**Affiliations:** 1 Research laboratory of Hepatobiliary Diseases, Second Xiangya Hospital of Central South University, Changsha 410011,China; 2 Department of Medicine, Stanford University School of Medicine, Stanford, California 94305, USA

**Keywords:** gallbladder neoplasms, gallbladder polyp, chronic cholecystitis, tumor-associated antigen, MK-1, regenerating gene Ⅳ, immunohistochemistry

## Abstract

**Background:**

To study the expression of MK-1 and RegⅣ and to detect their pathological significances in benign and malignant lesions of gallbladder.

**Methods:**

The expression of MK-1 and RegⅣ was detected by immunohistochemical method in paraffin-embedded sections of surgical resected specimens from gallbladder adenocarcinoma (n = 108), peritumoral tissues (n = 46), adenomatous polyp (n = 15), and chronic cholecystitis (n = 35).

**Results:**

The positive rate of MK-1 or RegⅣ expression was significantly higher in gallbladder adenocarcinoma than that in peritumoral tissues (χ^2^_MK-1 _= 18.76, *P *< 0.01; χ^2^_RegⅣ _= 9.92, *P *< 0.01), denomatous polyp (χ^2^_MK-1 _= 9.49, *P *< 0.01; χ^2^_RegⅣ _= 8.59, *P *< 0.01) and chronic cholecystitis (χ^2^_MK-1 _= 24.11, *P *< 0.01; χ^2^_RegⅣ _= 19.24, *P *< 0.01). The positive cases of MK-1 and/or RegⅣ in the benign lesions showed moderately- or severe-atypical hyperplasia of gallbladder epitheli. The positive rates of MK-1 were significantly higher in the cases of well-differentiated adenocarcinoma, no-metastasis of lymph node, and no-invasiveness of regional tissues than those in the ones of differentiated adenocarcinoma, metastasis of lymph node, and invasiveness of regional tissues in gallbladder adenocarcinoma (*P *< 0.05 or *P *< 0.01). On the contrary, the positive rates of RegⅣ were significantly lower in the cases of well-differentiated adenocarcinoma, no-metastasis of lymph node, and no-invasiveness of regional tissues than those in the ones of differentiated adenocarcinoma, metastasis of lymph node, and invasiveness of regional tissues in gallbladder adenocarcinoma (*P *< 0.05 or *P *< 0.01). Univariate Kaplan-Meier analysis showed that decreased expression of MK-1 (*P *= 0.09) or increased expression of RegⅣ (*P *= 0.003) was associated with decreased overall survival. Multivariate Cox regression analysis showed that decreased expression of MK-1 (*P *= 0.033) and increased expression of RegⅣ (*P *= 0.008) was an independent prognostic predictor in gallbladder adenocarcinoma.

**Conclusions:**

The expression of MK-1 and/or RegⅣ might be closely related to the carcinogenesis, clinical biological behaviors, and prognosis of gallbladder adenocarcinoma.

## Introduction

MK-1, also known as Ep-CAM, is a type-I transmembrane protein with cell adhesion activity expressed on normal epithelial cells of various tissues including stomach, colon, pancreas, lung, breast and ovary. MK-1 has been suggested to be involved in the differentiation and growth of epithelial cells under normal physiological conditions through its homotypic cell-cell adhesion activity [1-5]. Because it is over-expressed on most carcinomas, MK-1 has been used as a target for diagnosis and therapy of cancer [1-5]. The regenerating gene (Reg) family, a group of small secretory proteins, is involved in cell proliferation or differentiation in digestive organs, is upregulated in several gastrointestinal cancers, and functions as trophic or anti-apoptotic factors [6-11]. RegIV, a member of the regenerating gene family, is involved in digestive tract malignancies, including the stomach, colorectum, and pancreas, as well as in benign diseases such as ulcerative colitis [6-11]. RegIV overexpression in tumor cells has been associated with cell growth, survival, adhesion, and resistance to apoptosis [6-11]. Although the expression of MK-1 has been reported in carcinomas of various origins, only one study has described its expression in gallbladder adenocarcinoma. No study on the expression of RegIV in gallbladder adenocarcinoma has been published. In the present study, we studied the expression of MK-1 and RegⅣ in various benign and malignant lesions of gallbladder, and evaluated their prognostic usefulness.

## Materials and methods

### Patients and clinical information

A total of 204 specimens, including 108 adenocarcinomas, 46 peritumoral tissues, 35 chronic cholecystitis tissues, and 15 gallbladder polyp, were studied ethically with pre-approval from Ethics Committee of Human Study of Central South University. All of these samples were collected between 1996 and 2006. Among the 108 adenocarcinoma, 31 cases are male (28.7%) and 77 cases are female (71.3%) with an average age of 52.6 ± 11.2 years. Diagnoses of adenocarcinomas were based on morphological criteria, immunohistochemical staining, and the clinical findings. The histopathologic subtypes of the 108 adenocarcinomas include 36 well-differentiated adenocarcinomas (33.3%), 31 moderately-differentiated adenocarcinomas (28.7%), 30 poorly-differentiated adenocarcinomas (27.8%), and 11 mucinous adenocarcinomas (10.2%). The invasion was evaluated according to the standard criteria for T-stages [12]. Among the 108 adenocarcinomas, 14 cases are T1, 35 cases are T2, 37 cases are T3, and 22 causes are T4 stage. Invasion of gallbladder surrounding tissues and organs was found in 59 patients (54.6%) with adenocarcinoma while 59 patients had regional lymph node metastasis (54.6%). 58 cases had gallstones (53.7%). Surgery included radical resection for 34 adenocarcinoma (31.5%), palliative surgery for 48 adenocarcinomas (44.4%) and no resection operation for 26 cases (24.1%) (The specimens were obtained with surgical biopsy in these patients). Survival information of 67 cases among the 108 adenocarcinomas was obtained through letters and phone calls. Among them, 20 cases survived over 1 year and 47 cases survived less than 1 year. The chronic cholecystitis, gallbladder polyp and peritumoral tissues were diagnosed according to the published standard criteria [13]. The 46 peritumoral tissues (distance from cancer ≥ 3 mm) were obtained from the 108 patients with adenocarcinomas. Among them, 10 tissues are normal, 10 tissues are mild dysplasia, 12 tissues are moderate dysplasia, and 14 tissues are severe dysplasia. Among the 35 cases with chronic cholecystitis, 15 cases are males (42.9%) and 20 are females (57.1%). According to the criteria for dysplasia described by Dowling and Kelly [14], the 35 cases of chronic cholecystitis (15 have chronic cholecystitis only and 20 have chronic cholecystitis accompanied by gallbladder stone) were classified into normal and mild, moderate, and severe dysplasia: 11 cases without cellular atypia as normal mucosa, 12 cases with mild cellular atypia as mild dysplasia, 7 cases with moderate cellular atypia as moderate dysplasia, and 5 cases with severe cellular atypia as severe dysplasia. Among the 15 polyps, 10 were confirmed to have epithelial normal to mild dysplasia by pathological examination and 5 have moderate to severe dysplasia.

### EnVision immunohistochemistry

Four-micrometer-thick sections were cut from routinely paraffin-embedded tissues. Immunostaining was performed using a peroxidase-based EnVision™ Detection kit (Dako Laboratories, Carpinteria, CA, USA) following the user manual. The mouse anti-MK-1, rabbit anti-RegIV and HRP-conjugated second antibodies were purchased from Santa Cruz Biotechnology, Inc. **(**Santa Cruz, CA, USA).

### Pathological evaluation

Stained slides were evaluated in a blinded manner in all cases for membrane and cytoplasm staining. MK-1 and RegIV expression was defined as the presence of specific staining on the surface membranes and cytoplasm of tumor cells. MK-1 and RegIV expression was considered as positive when over 25% of tumor cells exhibited a positive reaction [13,15].

### Statistical analysis

Data was analyzed using the statistical package for the Social Sciences Version 13.0 (SPSS 13.0). The inter-relationship of MK-1 or RegIV expression with histology or clinical factors was analyzed using χ^2 ^or Fisher's exact test. Kaplan-Meier and log-rank test were used for univariate survival analysis. Cox proportional hazards model was used for multivariate analysis and to determine the 95% confidence interval. Results were considered significant if a two-sided *P *value of less than 0.05 was obtained.

## Results

### MK-1 and RegIV expression in benign and malignant lesions of gallbladder

**Figure 1:**
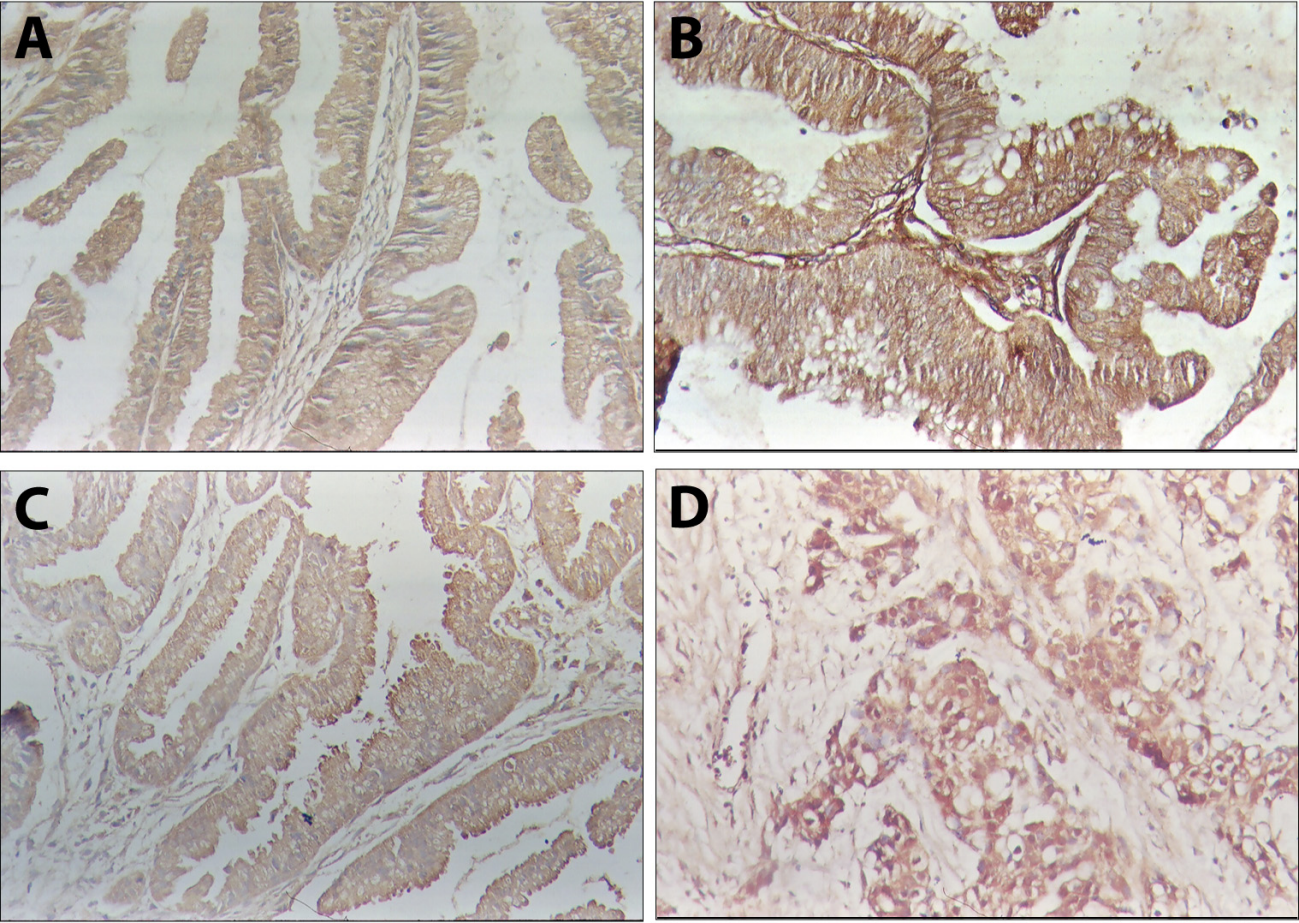
**Expression of MK1 and RegIV in the benign and malignant lesions of gallbladder**. EnVision immunohistochemistry, original magnification x200. MK1 and RegIV positive reaction was mainly localized in the cell membrance and/or cytoplasm. A, MK1 positive expression in peri-tumor tissue with moderate atypical hyperplasia. B, MK1 positive expression in highly differentiated gall bladder adenocarcinoma. C, RegIV positive expression in polyp with severe atypical hyperplasia. D, RegIV positive expression in moderately differentiated gall bladder adenocarcinoma.

The expression of MK-1 and RegIV was primarily located in the membrane and cytoplasm (Figure 1). The positive expression of MK-1 and RegIV in gallbladder adenocarcinoma samples was significantly higher than in peritumoral tissues, polyp or chronic cholecystitis (*P *< 0.01) (Table 1). Among the 108 gallbladder adenocarcinoma samples, 67 of them were MK-1 positive (62.0%) and 58 were RegIV positive (53.7%). The positively expressed peritumoral tissue, polyp and chronic cholecystitis showed atypical hyperplasia of epitheli.

**Table 1:** Relationship between ANXA1 and ANXA2 expression and the lifetime of gall bladder adenocarcinoma

Type	Case Number	MK-1			RegIV		
		
		Positive Cases (%)	χ^2^	*P*	Positive cases (%)	χ^2^	*P*
**Gall bladder adenocarcinoma**	108	67 (62.0)			58 (53.7)		

**Peri-tumor tissues**	46	11 (23.9)	18.76	< 0.01	12 (26.1)	9.92	< 0.01

**Gall bladder polyp**	15	3 (20.0)	9.49	< 0.01	2 (13.3)	8.59	< 0.01

**Chronic cholecystitis**	35	5 (14.3)	24.11	< 0.01	4(11.4)	19.24	< 0.01

Compared with Gall bladder adenocarcinoma

### MK1 and RegIV expression is associated with the clinicopathological characters of adenocarcinoma

In adenocarcinomas that were highly differentiated, with tumor diameter < 2 cm, with no lymph node metastasis and no peritissue invasion, MK-1 positive expression rate was significantly higher than those that were moderately- or low-differentiated, with tumor maximum diameter ≥ 2 cm, with lymph node metastasis and peritissue invasion (*P *< 0.05). The expression of RegIV showed opposite pattern (*P *< 0.05). The expression of MK-1 and RegIV showed no obvious relation with other clinicopathological characters, such as age, sex or cholecystolithiasis (*P *> 0.05) (Table 2).

**Table 2:** Relationship between MK-1 and RegIV expression and the clinical pathology characters of adenocarcinoma

Clinical Pathological Characters	Number of cases	MK-1			RegIV		
		
		Positive cases (%)	χ^2^	*P*	Positive cases (%)	χ^2^	*P*
**Sex**							
Male	24	16 (66.7)	0.28	> 0.05	11 (45.8)	0.77	> 0.05
Female	84	51 (60.7)			47 (56.0)		
							
**Age**							
≤ 45 yr	31	19 (61.3)	0.01	> 0.05	15 (48.4)	0.49	> 0.05
> 45 yr	77	48 (62.3)			43 (55.8)		
							
**Pathological type**							
Highly differentiated	36	29 (80.6)	16.35	< 0.01	12 (33.3)	9.94	< 0.05
Moderately differentiated	31	20 (64.5)			19 (61.3)		
Low differentiated	30	10 (33.3)			21 (70.0)		
Mucous adenocarcinoma	11	8 (72.7)			6 (54.5)		
							
**Maximal diameter**							
< 2 cm	31	23 (74.2)	2.73	> 0.05	13 (41.9)	2.42	> 0.05
≥2 cm	77	44 (57.1)			45 (58.4)		
							
**Lymphatic metastasis**							
None	49	39 (79.6)	11.74	< 0.05	20 (40.8)	4.24	< 0.05
Yes	59	28 (47.6)			38 (64.4)		
							
**Peritissue invasion**							
None	49	37 (75.5)	6.91	< 0.05	21 (42.9)	4.24	< 0.05
Yes	59	30 (50.9)			37 (62.7)		
							
**Cholecystolithiasis**							
None	50	28 (56.0)	1.44	> 0.05	24 (48.0)	1.22	> 0.05
Yes	58	39 (67.2)			34 (58.6)		

### Relationship between MK-1 and RegIV expression and the lifetime of patients with gall bladder adenocarcinoma

**Figure 2:**
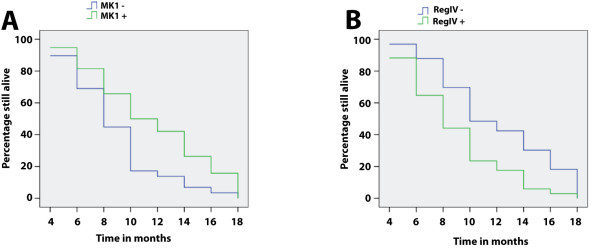
**MK1 and RegIV expression and survival in patients with adenocarcinoma of gallbladder**. A, Kaplan-Meier plots of overall survival in patients with gallbladder adenocarcinoma and with MK1 positive and negative expression. B, Kaplan-Meier plots of overall survival in patients with gallbladder adenocarcinoma and with RegIV positive and negative expression.

According to survey by mail or phone (2 years), we got 67 profiles from the 108 gallbladder adenocarcinoma, 20 of them survived more than or equal to 1 year after surgery, 47 of them had less than 1 year life cycle, and mean life cycle was 9.6 ± 5.2 months. Among the 67 cases, the positive expression of MK-1 and RegIV was 38 (56.7%) and 34 (50.7%). After Kaplan-Meier survival analysis, we found that the mean survival time after surgery was closely related to pathological types (*P *= 0.031), maximum diameter of tumor (*P *= 0.003), lymphatic metastasis status and peritissue invasion (*P = *0.002). The mean survival time after surgery was significantly higher in the MK-1 positive cases than the negative ones (*P *= 0.009), while the mean survival time after surgery was significantly lower in the RegIV positive cases than the negative ones (*P *= 0.003) (Figure 2). Table 3 summarizes the results of multivariate survival analysis using the Cox proportional hazards model. Multivariate analysis revealed that maximum diameter of tumor ≥ 2 cm, lymphatic metastasis and peritissue invasion, MK-1 negative expression and RegIV positive expression were negatively correlated with mean survival time after surgery, positively correlated with mortality, and are independent prognostic markers.

**Table 3:** Multivariate Cox regression analysis of the life cycle of 67 gall bladder adenocarcinoma patients

Group	Factors	RC^a^	SE^b^	RR^c^	*P value*	95% CI^d^	
						Lower	Upper
**Pathology type**	Adenomacanceration/well-/moderately-/poorly-differentiated/mucous adenocarcinoma	0.554	0.301	1.74	0.052	0.96	3.14
**Tumor diameter**	< 2.0 cm/≥ 2.0 cm	0.901	0.331	2.46	0.031	1.29	4.71
**Lympho node metastasis**	No/Yes	0.964	0.341	2.62	0.027	1.34	5.12
**Surrounding tissue invasion**	No/Yes	0.998	0.415	2.71	0.025	1.20	6.12
**MK-1**	-/+	-0.456	0.228	0.63	0.033	0.41	0.99
**RegIV**	-/+	1.153	0.475	3.17	0.008	1.25	8.04

## Discussion

Our study shows the prognostic significance of MK-1 and RegIV expression as a tumor biological marker in patients with gallbladder adenocarcinoma. We found that positive expression rate of MK-1 and RegIV were higher in gallbladder adenocarcinoma than those in peritumoral tissues, polyp and chronic cholecystitis. Statisitical analysis revealed that MK-1 negative expression and RegIV positive expression is negatively correlated with mean survival time after surgery, positively correlated with mortality, and are independent prognostic markers.

The expression of MK-1 has been previously reported in several carcinomas. In gastric carcinomas, MK-1 is more frequently expressed in cardiac tumors (with 50% expression rate), in large (> 3 cm) tumors, and in specimens from patients with more than five metastatic lymph nodes [2]. In urinary bladder carcinoma, 56.8% were positive for MK-1 protein expression and significant correlations were observed between MK-1 expression and tumor grade, schistosoma, DNA ploidy and tumor recurrence [3]. In Gallbladder carcinoma, expression of MK-1 was found in 50 (79%) of 63 tumor samples. Multivariate analysis showed that MK-1 expression was an independent prognostic marker, and Kaplan-Meier curves showed that MK-1 expression was significantly related to increased overall survival, suggesting that MK-1 expression is a prognostic marker in gallbladder carcinoma [4]. In our study the positive expression rate of MK-1 was slightly lower than what was previously reported (62% vs 79%), however, our statistical analysis revealed similar prognostic significance of MK-1 expression.

The expression of RegIV has also been reported to be increased in some carcinomas including prostate, pancreatic and gastric cancer [6-11]. The expression of RegIV has been found to be related to the carcinogenesis, clinical biological behaviors, and prognosis of the carcinomas studied. Most of the high RegIV cases have poorly differentiated, high clinical stage, prone to metastasis and strong invasion ability, which are all considered being bad indicators of cancer prognosis [6-11,16]. These results all support that RegIV may involve in cancer formation and affect its development and prognosis. To the best of our knowledge our study is the first of RegIV expression in gallbladder adenocarcinoma. We showed that the positive expression of RegIV in gallbladder adenocarcinoma samples was significantly higher than in benign gallbladder lesions. In adenocarcinomas, MK-1 positive expression rate was significantly higher in samples that were highly differentiated, with tumor diameter < 2 cm, with no lymph node metastasis and no peritissue invasion. Moreover, RegIV positive expression was negatively correlated with mean survival time after surgery, positively correlated with mortality, and are independent prognostic markers. These findings indicate that RegIV is a promising tumor marker in gallbladder adenocarcinoma.

## Competing interests

The authors declare that they have no competing interests.

## Authors' contributions

LY and ZY designed the study, and performed the experiment and statistical analysis and drafted the manuscript. SL participated in the immunostaining. JL participated in the statistical analysis. All authors read and approved the final manuscript.
